# Two Handheld Retinograph Devices Evaluated by Ophthalmologists and an Artificial Intelligence Algorithm

**DOI:** 10.3390/jcm13226935

**Published:** 2024-11-18

**Authors:** Pedro Romero-Aroca, Benilde Fontoba-Poveda, Eugeni Garcia-Curto, Aida Valls, Julián Cristiano, Monica Llagostera-Serra, Cristian Morente-Lorenzo, Isabel Mendez-Marín, Marc Baget-Bernaldiz

**Affiliations:** 1 Ophthalmology Service, University Hospital Sant Joan, Institut de Investigació Sanitaria Pere Virgili (IISPV), Universitat Rovira & Virgili, 43204 Reus, Spain; eugenigorg@gmail.com (E.G.-C.); monicallagostera@hotmail.com (M.L.-S.); cristian_ml87@outlook.com (C.M.-L.); mariaisabel.mendez@salutsantjoa.cat (I.M.-M.); mbaget@gmail.com (M.B.-B.); 2 Responsible for Diabetic Retinopathy Eye Screening System in Primary Care in Baix Llobregat Barcelona (Spain), Institut de Investigació Sanitaria Pere Virgili (IISPV), Universitat Rovira & Virgili, 43204 Reus, Spain; bfontoba@gmail.com; 3 ITAKA Research Group, Department of Computer Science and Mathematics, Universitat Rovira & Virgili, 43007 Tarragona, Spain; aida.valls@urv.cat (A.V.); julianefren.cristiano@urv.cat (J.C.)

**Keywords:** artificial intelligence, diabetic retinopathy, handheld retinal camera, public health, screening, smartphones, telemedicine, image quality

## Abstract

**Background/Objectives:** Telemedicine in diabetic retinopathy (RD) screening is effective but does not reach the entire diabetes population. The use of portable cameras and artificial intelligence (AI) can help in screening diabetes. **Methods:** We evaluated the ability of two handheld cameras, one based on a smartphone and the other on a smartscope, to obtain images for comparison with OCT. Evaluation was carried out in two stages: the first by two retina specialists and the second using an artificial intelligence algorithm that we developed. **Results:** The retina specialists reported that the smartphone images required mydriasis in all cases, compared to 73.05% of the smartscope images and 71.11% of the OCT images. Images were ungradable in 27.98% of the retinographs with the smartphone and in 7.98% with the smartscope. The detection of any DR using the AI algorithm showed that the smartphone obtained lower recall values (0.89) and F1 scores (0.89) than the smartscope, with 0.99. Low results were also obtained using the smartphone to detect mild DR (146 retinographs), compared to using the smartscope (218 retinographs). **Conclusions:** we consider that the use of handheld devices together with AI algorithms for reading retinographs can be useful for DR screening, although the ease of image acquisition through small pupils with these devices needs to be improved.

## 1. Introduction

Diabetic retinopathy (DR) can develop from diabetes mellitus (DM). It causes retinal vasculature in the form of microangiopathy and, at the same time, affects the neurons involved. Consequently, DR is a neurovascular complication of the retina [1].

DM currently affects 537 million patients worldwide, and this number is predicted to rise to 643 million by 2030 and to 783 million by 2045 [2]. The increase in DR runs parallel to this, as DR currently affects 6.4 million people in Europe alone, with an expected increase to 8.6 million by 2025, of whom 30% will need treatment [3]. DR also remains the leading cause of preventable vision loss and blindness in adults aged 20 to 74 years, especially in middle- and high-income countries [4,5].

Given the rapid global increase in DM and increasing life expectancy, DR will remain a major public health challenge. Screening for DR through telemedicine has proven to be effective [6]; therefore, it should be promoted where it is already in place and implemented where it is not. Only early detection of DR will allow us to both prevent its evolution to more severe forms and to treat it early to avoid vision loss [7].

Current screening systems can reach a significant proportion of the patients with DM, but it is difficult to screen all patients every one or two years as recommended [2]. In our experience, only 40% of patients are screened annually [8], despite having four non-mydriatic camera units in our health area. To improve this, extending screening to every two years, according to the metabolic control of patients, has been proposed, along with bringing in other professionals—such as general practitioners (GPs), endocrinologists, and paediatricians—into the system. At a technical level, the use of portable cameras has been proposed. These cameras, together with artificial intelligence (AI) algorithms that read retinographs automatically and help to detect DR, can be used by GPs [9,10].

Based on these proposals, the present study evaluated two portable cameras developed by our team to detect DR, and the results were compared. In addition, a comparison between the retinographies of the three devices was carried out using an AI algorithm [11].

## 2. Materials and Methods

### 2.1. Setting and Design

This study was based on a real population of patients with type 2 DM. The reference was our health area (Hospital Universitari de Sant Joan de Reus, Spain) of 226,508 inhabitants, of whom 17,792 patients are registered with DM. The study has been ongoing, based on our diabetic retinopathy screening program [12].

### 2.2. Objectives

The aim of this study was to evaluate the possibility of using portable cameras instead of fixed non-mydriatic cameras for diabetic retinopathy screening. To achieve this, we compared two portable cameras: one based on a smartphone system (VISTAVIEW, Topcon Healthcare^®^, USA) and a second that uses a classic but portable retinography system and the AURORA retinograph (AURORA™, Optomed Topcon^®^, Japan) as a smartscope. Once the sample collection was finished, we compared the retinographs obtained with the smartphone (smartscope) and the retinographs obtained with the standard capture system that we use in the screening program, which uses the OCT TRITON^®^ (TOPCON^®^, Japan) to obtain retinographies.

In a second phase, the retinographs obtained using the two portable cameras and the OCT retinograph were transferred to electronic medical records (EMRs). From the medical record, the images were analysed using the AI system that we developed in the hospital (MIRA©) [11,12,13,14,15], comparing the results obtained with the images obtained from the three systems.

### 2.3. Inclusion Criteria

Inclusion criteria were as follows: patients with type 2 DM from our DR screening programme.

### 2.4. Exclusion Criteria

Exclusion criteria were as follows: type 1 DM and other causes of diabetes mellitus, such as gestational DM, and retinographs that were not in a patient’s electronic medical record or that did not use the DICOM format.

### 2.5. Methods

For each patient, a retinograph of each eye was obtained from the three devices (the two portable devices and the standard equipment). The images were focused on a point located between the fovea and the temporal side of the papilla, with retinographs using 45–55°. Pharmacological mydriasis was achieved by applying tropicamide eye drops if necessary. The retinographs were sent via a PACS (picture archiving and communication system) to the patient’s electronic medical record (EMR), where they were stored in DICOM (Digital Imaging and Communications in Medicine), a standard format that guarantees the interoperability and compatibility of medical images.

Table 1 shows DR classified with the international classification of DR [16] at ‘Level 1 or mild DR’ and ‘Level 2 or moderate DR’. At ‘Level 3 or severe DR’, we introduced a variant to include images with new vessels in the retina (proliferative DR), and we included any patients with macular oedema. Therefore, our Level 3 would be equivalent to a vision-affecting referable DR or VTDR (vision-threatening diabetic retinopathy).

### 2.6. Technical Characteristics of the Three Devices

The cameras used were as follows: first, the standard one with which we carry out the DR screening, which is the Oct TRITON, from which the retinographies read by the ophthalmologist responsible for the screening are obtained; second, the portable VISTAVIEW camera, which uses an Android-type smartphone; and third, the portable AURORA camera, which is a portable retinograph or smartscope. The technical difference between them is limited to the pixelation of the images which, for the OCT TRITON and the portable AUROA, is 5 Mpixels and, for the VISTAVIEW smartphone, is 28.4 Mpixels.

### 2.7. Ethics and Consent

The study was carried out with the approval of the local ethics committee, the Medical Research Ethics Committee (CEIM) of the Pere Virgili Health Research Institute (IISPV), Tarragona, Spain, RetinaReadRisk approval code, protocol version 1. 10 March 2022, CEIM reference number: 071/2022.

This ethical consent was signed by all patients and was the same for the two funding entities. The project received co-funding from the European Institute of Innovation and Technology (EIT), from the European Union under grant agreements 220718 and 230123, and from the Instituto de Salud Carlos III (ISCIII) through the project PI21/00064 (co-funded by the European Union).

### 2.8. Statistical Methods

Data were analysed via the SPSS program, version 22.0 (IBM^®^ Statistics, Chicago, IL, USA). A descriptive statistical analysis of the quantitative data was performed by determining the mean and standard deviation. For qualitative data, frequency and percentage analysis in each category were used. For all parameters, descriptive statistics (including number of values, mean, standard deviation (SD), and standard error of mean (SEM)) were calculated. A *p*-value less than 0.05 was considered statistically significant.

The screening performance of the AI Machine Reading Algorithm (MIRA)© was measured using a 2 × 2 confusion matrix/contingency table for each team. Given a classified dataset, there were four basic combinations of real and assigned: true positives (TP), true negatives (TN), false positives (FP), and false negatives (FN).

Statistical evaluation of the dataset included sensitivity, specificity, positive predictive value or accuracy, negative predictive value, harmonic mean or F1 score, and accuracy.

Sensitivity or recall is the proportion of the population for which the test is correct and, in the context of clinical diagnosis, sensitivity and specificity values are considered good when they exceed 80%. Positive or accuracy predictive value, on the one hand, and negative predictive value provide a proportion of the population with a given test result for which the test is correct or incorrect, respectively.

To assess concordance, we determined the values of accuracy and harmonic mean. Diagnostic accuracy is expressed as the proportion of correctly classified subjects among all subjects. Accuracy values of >0.8 show an almost perfect correlation, while values of 0.6–0.79 imply a significant correlation, 0.4–0.59 a moderate correlation, 0.2–0.39 a regular correlation, and 0–0.2 a poor correlation. The harmonic mean or F1 score, also known as the Sørensen–Dice coefficient or Dice similarity coefficient (DSC), is a measure of test performance that combines precision (the positive predictive value) and recall (sensitivity). The highest possible value of F1 is 1, indicating perfect accuracy and recall, and the lowest possible value is 0 if the accuracy or recall is zero. The advantage of using the harmonic mean over accuracy is that it is not affected by the prevalence of the disease, which is the case for accuracy; thus, if the prevalence is low, it tends to provide high accuracy values [17].

## 3. Results

### 3.1. A Retinograph of Each Eye Was Obtained with Each of the Three Devices

In total, 4260 retinographs were obtained from 2130 patients. The mean age of the sample was 67.43 ± 11.21 years (38–91 years), of which 1256 (58.96%) were women. Regarding the treatment of DM, 264 patients (12.39%) were treated through diet alone, 1810 (84.98%) with oral antidiabetics, and 56 (2.63%) with insulin or insulin + oral antidiabetics. Finally, 1527 (71.69%) had high blood pressure.

Table 2 shows the differences between the retinographs across the three devices. We first tried to obtain the retinographs without pupil dilatation, but the images did not show any form of DR in 69.83% with the VISTAVIEW, in 83.91% of the cases with the AURORA, and in 71.11% of cases with the Triton.

After dilation, the percentage of images that were not of sufficient quality for ophthalmologists to evaluate was 27.98% with the VISTAVIEW, 7.98% with the AURORA, but 0.93% with the Triton. The principal causes of poor quality were the following:

(i) The difficulty in focusing the handheld device, particularly the VISTAVIEW;
(ii) The presence of cataracts, obviously affecting all three devices equally;
(iii) Slight mydriasis.

Patients with moderate DR or severe DR were identified with similar success across all three devices, but for mild DR, the AURORA and the Triton were more successful than the VISTAVIEW.

### 3.2. Statistical Analyses

Data comparing the handheld devices with the Triton are presented in Table 3. Applying the 2 × 2 contingency table, we found the two handheld devices to be reliable. Although the VISTAVIEW had a somewhat lower score in the application of the F1 statistic or harmonic mean (0.93 for any DR and 0.91 for mild DR), the results were good for both in any case.

### 3.3. The Comparative Study of the Results of the Three Devices Using the MIRA© Algorithm

Table 4 shows the results from reading the images obtained with the MIRA© automatic-reading AI algorithm.

According to the type of DR, the MIRA© algorithm read retinographs from the AURORA and Triton devices more accurately than those from the VISTAVIEW. Mild DR was detected in fewer cases with the VISTAVIEW (102 cases) compared to the AURORA (218) and the Triton (220). The latter are what we consider real cases, as they were identified by retina specialists who are the gold standard. The AI algorithm identified moderate DR and severe DR with similar success across the three devices.

### 3.4. Statistical Study of the Results Obtained by AI Algorithm MIRA©

We evaluated the images obtained by the three devices—the Triton tomography being what we currently consider to be the gold standard—with images read by specialist ophthalmologists.

Table 5 shows the results of the statistical analysis of the performance of the three devices in the detection of DR, using 2 × 2 contingency tables. The sensitivity of the VISTAVIEW smartphone to identify any DR was 0.89; it was outperformed by the portable AURORA (0.99) and by the images obtained by the Triton OCT (0.99).

Similarly, for mild DR, the scores for the VISTAVIEW dropped to a sensitivity of 0.83, with a positive predictive value of 0.85, compared to the values of 0.99 sensitivity for both the AURORA and the Triton and a positive predictive value of 0.98 for the AURORA. This is confirmed by the TP values and by the F1 harmonic average score: the smartphone scored a harmonic average of 0.89 for detecting any DR and 0.83 for mild DR.

In summary, the statistical study indicates that the AURORA and Triton OCT devices detect with high reliability when DR is present or absent at all stages of DR. The VISTAVIEW device is safe when it indicates that there is no DR but unreliable in detecting cases of mild DR.

## 4. Discussion

The constant increase and diversity in the population with DM has led to the pursuit of new ways of reaching as much of the diabetic population as possible, and various publications by scientific societies have reported progress via telemedicine [2,6].

The authors believe that portable devices used together with AI algorithms that can read retinographs will be able to reach a larger population than the current model allows [6,18,19,20,21,22].

The present study aimed to evaluate the usefulness of two of those portable devices in screening patients with diabetes mellitus. We selected one smartphone camera and a smartscope or portable retinoscope. We compared the images obtained with these two devices with the retinography obtained through Triton optical coherence tomography, which is the standard for DR screening in our health area. The results showed that the smartphone produced a higher number of ungradable retinographs (27.98%) than the AURORA smartscope (7.98%) or the standard Triton OCT (0.93%). We can report that mydriasis was necessary for all patients when using the smartphone compared to 73.05% using the AURORA and 71.11% using the Triton. The major difficulty was being able to focus accurately on the retina when using the smartphone. We can also report that the smartphone had more difficulty in distinguishing mild DR forms (only 93 cases) than the AURORA (109 cases) and the Triton (110 cases).

In a second step, we analysed the results after the automatic reading of retinographs using the AI algorithm developed in our hospital. As in the first step, the automatic image analysis detected fewer cases of retinographies with mild DR when they were obtained by the VISTAVIEW device compared to the other two devices. Thus, only 146 retinographs with mild DR were detected, compared to 218 with the AURORA or 220 with the OCT.

Regarding the difference between automatic image reading by the AI system (MIRA) and retinologists, we can see the comparison in Table 4. In this table, it is seen that the differences between automatic and human assessments are mainly at the level of gradability; the smartphone has many no-gradability images (31.97%) compared to 8.02% in the smastcope in AI reading, while when reading by retinologists, the no-gradability is only 0.93%.

Regarding the detection of diabetic retinopathy, the differences between human and automatic systems are minimal, so the automatic system for the smartphone detects 7.88% of images with any DR, the smartscope detects 8.54%, while the human eye detects 8.82%. If we look at the level of severe diabetic retinopathy, which would be the cases in which detection is essential, both the human eye and the AI detect all cases in total; the 36 retinography affected by severe diabetic retinopathy are detected.

We could conclude that, for both the human eye and the AI algorithm, the detection of mild DR is more difficult with the smartphone. Perhaps the difference in pixelation of the images between devices may be the cause, or perhaps it is the difficulty in obtaining clear images with the smartphone.

The statistical analysis revealed that the sensitivity or recall, and the positive predictive value or precision, were poorer for the images obtained with the smartphone for detection of any DR or mild DR. The differences were less marked when using the harmonic mean or F1 score, which scored the VISTAVIEW smartphone at 0.97 for accuracy and the AURORA smartscope at 0.99 for the presence of any DR or mild DR. However, when applying the harmonic average, the values changed: 0.93 and 0.91 for the VISTAVIEW and 0.98 and 0.95 for the AURORA for any DR and mild DR, respectively. To differentiate between the smartphone and the smartscope, we had to calculate the harmonic mean or F1 score. This is necessary when prevalence is very low, as was the case in our sample (9%).

In the literature, a similar study was carried out by Jacoba et al. [23]. This study involved a clinical series on 225 eyes of 116 patients in which the presence of macular pathology alone was evaluated. That study used two portable retina smartscopes, the AURORA® (TOPCON, Japan) and the RetinaVue™ (Wellch Allyn, USA). Comparing them with the Cirrus™ OCT (Zeiss, Germany) camera, the study reported good specificity but low sensitivity of the portables in detecting maculopathy.

Another similar study aimed not only to screen for DR, but also to detect macular pathology, whether diabetic or not [22]. The study reported ungradability with the OCT at 0.9%, a value similar to ours, and 4.4% with the AURORA, a value somewhat lower than ours (i.e., 7.98%), although the focus was on the macular area rather than on the whole retina.

A review of the efficacy of smartphones via a meta-analysis carried out by Tan et al. [20] reported the results obtained with five Android or iPhone5S mobile devices. The results showed that, for the detection of any DR, mean sensitivity was 87% (minimum 74%, maximum 94%) and specificity was 94% (minimum 81%, maximum 98%), values not dissimilar to those we obtained using the VISTAVIEW (sensitivity of 88% and specificity of 99%); although, notably, sensitivity for mild DR was very low, with an average of 39% (minimum 10%, maximum 79%). It is important to consider that smartphone technology is constantly changing and improving [24].

The AURORA smartscope has been tested in various studies, yielding results similar to ours. The largest sample used was that of Salongcay et al. in 2023 [25], who reported a percentage of unreadable images of 7.5% (similar to ours, with 7.07%) and an agreement of 82.4% (lower than ours, at 98%). Other studies have reported similar values to ours, with growth figures of 93% reported by Kubin et al. [26] and a positive predictive value of 98% and a specificity of 97% reported by Salongcay et al. [27].

One of the strengths of the present study is that the analysis of images was carried out using those found in the electronic medical record (EMR), rather than those stored in the equipment. This brings together the images in DICOM format for all devices and allows us to compare them more easily. Another strength is the much larger sample size compared to most previous studies. We were able to include 4260 images from 2130 patients: only the Salongcay study of 2023 included a higher number, with 5585 images from 2793 patients.

One limitation of the study is the use of only two devices, which hinders the extrapolation of the results to other devices. Furthermore, the use of a single retinograph focused on a point between the macula and temporal sides of the papilla might also limit the detection of DR, compared to studies that included more retinographs of more fields or perhaps wide-field retinographs, which have been shown to change the severity of DR [28]. Finally, another limitation is that, at Level 3 of DR, we included patients with proliferative retinopathy and macular oedema, which might alter the results when compared to other studies, although our findings can be used as a reference for studies that aim to evaluate the number of patients we detected with referable DR.

## 5. Conclusions

We can affirm that image capture systems or handheld retina cameras are useful for detecting DR in screening programmes and might be further aided through the incorporation of AI algorithms. We found that images captured with a smartscope or portable retinograph device are superior to those captured with a smartphone, which produced many unreadable images. Both devices require more efficient focusing in order to make image centring easier, improve image readability, and improve the quality of images obtained through small pupils to 3 mm, reducing the need for mydriasis.

## 6. Patents

Software MIRA 1.0, register SAFE CREATIVE code 2007104712196. Date and time: 10 July 2020, 11:24 UTC.

## Figures and Tables

**Table 1:** Classification used in this study. Level 3 includes severe DR, proliferative DR, and macular oedema. DR = diabetic retinopathy, DME= diabetic macular edema.

Level	Description
0	No retinopathy.
1	Mild DR. Microaneurysms only.
2	Moderate DR. More than just microaneurysms but less than severe.
3	Severe DR. Any of the following: more than 20 intraretinal haemorrhages in the 4 quadrants; venous beads defined in 2 quadrants; intraretinal microvascular abnormalities in 1 quadrant. Proliferative retinopathy. If DME is present, it will be included in Level 3.
Levels of DME included in Level 3 of current study	Mild DME: Some thickening of the retina or hard exudates at the posterior pole but distant from the centre of the macula. Moderate DME: thickening of the retina or hard exudates that approach the centre of the macula but do not involve the centre. Severe DME: thickening of the retina or hard exudates affecting the centre of the macula.

**Table 2:** Comparative study of the two portable devices against the evaluation by two retina specialists. % * = percentage of the total number of patients with diabetic retinopathy.

	Patients	Total Retinographs	Patients Who Required Mydriasis	Ungradable Retinographs	
VISTAVIEW	2130	4260	All cases	1192/27.98%	
AURORA	2130	4260	1556/73.05%	340/7.98%	
Triton	2130	4260	1536/71.11%	20/0.93%	
	No DR	Any DR	Mild DR	Moderate DR	Severe DR
VISTAVIEW	1962/69.83%	168/7.88%	93/55.37% *	57/33.92% *	18/10.71% *
AURORA	1948/83.91%	186/8.54%	109/58.39% *	59/31.72% *	18/9.89% *
Triton	1948/90.03%	188/8.82%	110/58.51% *	60/31.91% *	18/9.57% *

**Table 3:** Study of patients with and without DR. TN = true negative, FN = false negative, TP = true positive, FP = false positive, S = sensitivity, SP = specificity, TPV = true predictive value, NPV = negative predictive value, F1 score or mean harmony, and ACC = accuracy.

		TN	FN	TP	FP	S	SP	TPV	NPV	ACC	F1 Score
Any DR	VISTAVIEW	1400	20	168	24	0.89	0.98	0.99	0.99	0.97	0.93
	AURORA	1942	2	188	6	0.99	0.99	0.97	0.99	0.99	0.98
		TN	FN	TP	FP	S	SP	TPV	NPV	ACC	F1 score
Mild DR	VISTAVIEW	1404	17	93	20	0.85	0.99	0.82	0.99	0.93	0.91
	AURORA	1942	1	109	8	0.99	0.99	0.93	0.99	0.99	0.95

**Table 4:** The analysis of the images entered into the MIRA© automatic-reading algorithm.

Classification of Retinographies						
	Ungradable or MIRA	No DR	Any DR	Mild DR	Moderate DR	Severe DR
VISTAVIEW	1362/31.97%	2330/54.46%	284/7.88%	146/55.37%	102/33.92%	36/10.71%
AURORA	342/8.02%	3174/74.5%	372/8.54%	218/58.39%	118/31.72%	36/9.89%
Standard method	40/0.93%	3468/81.4%	376/8.82%	220/58.52%	120/31.91%	36/9.57%

**Table 5:** Statistical analysis using the AI algorithm. TN = true negative, FN = false negative, TP = true positive, FP = false positive, S = sensitivity, SP = specificity, TPV = true predictive value, NPV = negative predictive value, F1 score or mean harmony, and ACC = accuracy.

		TN	FN	TP	FP	S	SP	TPV	NPV	ACC	F1 Score
Any DR	VISTAVIEW^®^	2614	30	284	34	0.89	0.99	0.90	0.99	0.97	0.89
	AURORA^®^	1201	2	130	1	0.99	0.99	0.98	0.99	0.99	0.98
	Triton	3842	2	376	2	0.99	0.99	0.99	0.99	0.99	0.99
		TN	FN	TP	FP	S	SP	TPV	NPV	ACC	F1 score
Mild DR	VISTAVIEW^®^	2614	25	146	30	0.83	0.99	0.85	0.99	0.98	0.83
	AURORA^®^	1201	2	130	1	0.99	0.99	0.98	0.99	0.99	0.98
	Triton	3842	2	220	2	0.99	0.99	0.99	0.99	0.99	0.99

## Data Availability

The original contributions presented in this study are included in the article; further enquiries can be addressed to https://www.iispv.cat/cas-dxit/retinareadrisk-rrr/ (accessed on 28 October 2024) or directly to the authors. The data used, both in the database and in the training dataset for the AI systems, are kept at the following address https://www.iispv.cat/ (accessed on 28 October 2024) and can be accessed by the ophthalmology research group.
